# Predicting acute coronary syndrome in males and females with chest pain who call an emergency medical communication centre

**DOI:** 10.1186/s13049-019-0670-y

**Published:** 2019-10-17

**Authors:** Paul-Georges Reuter, Catherine Pradeau, Samantha Huo Yung Kai, Thibault Lhermusier, Arnaud Bourdé, Eric Tentillier, Xavier Combes, Vanina Bongard, Jean-Louis Ducassé, Sandrine Charpentier

**Affiliations:** 1 Emergency Department, Toulouse University Hospital, 31000 Toulouse, France; 2 UMR 1027, Paul Sabatier University Toulouse III, Inserm, Toulouse, France; 3 SAMU 92, Assistance Publique–Hôpitaux de Paris, Hôpital Raymond Poincaré, 92380 Garches, France; 4 SAMU 33, CHU de Bordeaux, 33076 Bordeaux cedex, France; 5 Unité de Soutien Méthodologique à la Recherche (USMR), Centre Hospitalier Universitaire de Toulouse (CHU de Toulouse), Toulouse, France; 6 Department of Cardiology, Rangueil University Hospital, Toulouse, France; 7 Department of Emergency, CHU de la Réunion, allée des Topazes, Université de la Réunion, 97400 Saint Denis, France

**Keywords:** Chest pain, Acute coronary syndrome, Emergency medical communication Centre, Accuracy, Sex disparity

## Abstract

**Background:**

Chest pain is a frequent reason for calls in emergency medical communication centre (EMCC). Detecting a coronary origin by phone is a challenge. This is especially so as the presentations differ according to gender. We aimed to establish and validate a sex-based model to predict a coronary origin of chest pain in patients calling an EMCC.

**Methods:**

This prospective cohort study enrolled patients at 18 years of age or older who called the EMCC because of non-traumatic chest pain. The main outcome was the diagnosis of acute coronary syndrome (ACS) determined by expert evaluation of patient files.

**Results:**

During 18 months, 3727 patients were enrolled: 2097 (56%) men and 1630 (44%) women. ACS was diagnosed in 508 (24%) men and 139 (9%) women. For men, independent factors associated with an ACS diagnosis were age, tobacco use, severe and permanent pain; retrosternal, breathing non-related and radiating pain; and additional symptoms. The area under the receiver operating characteristic curve (AUC) was 0.76 (95% confidence interval [CI] 0.73–0.79) for predicting ACS. The accuracy of the male model to predict ACS was validated in a validation dataset (Hosmer-Lemeshow test: *p* = 0.554); the AUC was 0.77 (95%CI 0.73–0.80). For women, independent factors associated with an ACS diagnosis were age ≥ 60 years, personal history of coronary artery disease, and breathing non-related and radiating pain. The AUC was 0.79 (95%CI 0.75–0.83). The accuracy of the female model to predict ACS was not validated in the validation dataset (Hosmer-Lemeshow test: *p* = 0.035); the AUC was 0.67 (95%CI 0.60–0.74).

**Conclusions:**

Predictors of an ACS diagnosis in patients calling an EMCC for chest pain differ between men and women. We developed an accurate predictive model for men, but for women, the accuracy was poor.

**Trial registration:**

This study is registered with ClinicalTrials.gov (NCT02042209).

## Background

Acute coronary syndrome (ACS) is a frequent pathology worldwide. Management consists in coronary reperfusion, by percutaneous intervention or fibrinolysis. The worst evolution consists in ventricular fibrillation and cardiac arrest. Survival depends on the total ischemic time: delay between chest pain onset and reperfusion [1]. The main symptom is chest pain or discomfort [2]. The first link for out-of-hospital chain of survival is patient education to call the emergency medical communication centre (EMCC) in case of chest pain. The second is the ability of the dispatcher, medical or not, to identify patients with ACS and to send an ambulance (with an emergency physician or not, depending on the country).

In a Copenhagen EMCC, chest pain was the second identified reason after minor trauma for calls, with 11% of the requests [3]. The sex ratio was 1:1 and over half of the patients were over 65 years of age [4]. When a contact occurred with an EMCC, the prevalence at 30 days of ACS ranged from 12 to 16% [5, 6]. For patients with ACS, the use of the EMCC was in the range of 23 to 43% [7, 8]. Patients with ST elevation myocardial infarction (STEMI) were given the highest priority in 82% of cases [9].

ACS diagnosis depends on electrocardiography (ECG) findings and biomarkers. At an EMCC, these data are not available. Only patient history and characteristics of chest pain can be investigated. A persistent pain or a typical location with radiation without associated symptoms influences the dispatcher to send a Mobile Intensive Care Unit (MICU) in France [10]. In Sweden, the intensity, the localisation of the pain and a history of ischemic heart disease were associated with the final diagnosis of ACS [11]. The accuracy of a computer-based decision support was compared with dispatchers’ decisions to predict ACS [5]. Sensitivity was greater with the computer- than dispatcher-based decision (90% vs. 83%), and that under triage (false negative) was 10 and 17%, respectively. The factors correlated to under triage were the time of the call (lunch time) and the level of medical knowledge of the dispatcher (assistant nurse versus dispatcher with no medical training) [12]. These findings confirm the need for a decision support tool to help dispatchers identify patients at risk of ACS.

Atypical clinical presentations are difficult to diagnose [13]. In older and diabetic patients, chest discomfort can be absent [14, 15]. Males and females also differ in clinical presentation of ACS [2]. Women, particularly those < 55 years old, most often describe atypical chest pain, such as discomfort, pinching, or burning, [16–20]. These differences in initial presentation lead to an increase in mortality among women (10% versus 5%) [21]. Total ischemic time is longer in women than men [8, 9, 22]. One explanation is a lower prioritization for women when calling call centres (79% for women and 89% for men) [9]. These results suggest that attention should be paid to recognize these patients as soon as possible. In creating a “by-phone” predictive score of ACS, items should differ according to sex.

We aimed to establish and validate a model to predict ACS for men and women calling an EMCC from information that can be recorded by phone.

## Methods

### Study design and setting

The DOREMI 2 prospective cohort study was conducted in three French university hospitals. This study is a follow-up to DOREMI 1, which was a pilot and feasibility study. The three participating EMCCs were located in Toulouse, Bordeaux and Saint-Denis de la Réunion. In 2017, the EMCCs served 1.318, 1.506 and 0.843 million inhabitants, respectively. After an evaluation by a dispatcher assistant, a physician manages every call for medical reasons. Depending on the dispatcher prioritization, calls are handled by a general practitioner or an emergency physician. In France, calls from patients with chest pain or discomfort are generally transferred to the emergency physician dispatcher. In response to a call, a medical dispatcher can give medical advice, recommend going into a medical care structure or send an ambulance, fire brigade, physician or MICU (ambulance with an emergency physician on board).

### Selection of participants

From May 2010 to November 2011, we consecutively included adults at 18 years of age or older who called the EMCC for non-traumatic chest pain. The “non-traumatic” characteristic was verified directly by asking the patient. The exclusion criterion was any difficulty in communicating: uncommunicative patient, language barrier, or inability to speak with the patient.

### Measurements

At the first call to the EMCC, the emergency physician recorded patient characteristics, cardiovascular risk factors, medical history and clinical presentation on a standardized form (Additional file 1). Follow-up data were collected 30 days after the call (D30) by telephone interview. A research assistant contacted the patient’s general practitioner and/or the patient directly. The patient was contacted in case of non-response from the general practitioner or in case of incomplete information.

At the D30 follow-up, the research assistant retrieved reports from the emergency department, hospitalization and additional examinations. They collected data on major adverse cardiovascular events (rehospitalisation, myocardial infarction, urgent revascularization or death), admission or a consultation in a cardiology unit, non-invasive imaging (transthoracic echocardiography, stress imaging with exercise or drug, cardiac MRI), coronary angiography and the final diagnosis during hospitalization. The final diagnosis of ACS was based on these data.

### Outcome

The outcome was a diagnosis of ACS by experts according to current guidelines [23].

STEMI was defined by the onset of a persistent ST-elevation on ECG, considered suggestive in the following cases: 1) at least two continuous leads with ST-segment elevation > 0.2 mV in leads V1-V3 or > 0.1 mV in leads V4-V9, V3R and V4R or 2) left bundle branch block with the presence of concordant ST-segment elevation.

Non–STEMI was diagnosed with compatible clinical presentation and ECG abnormalities in two continuous leads such as ST-segment depression or T-wave changes and elevated cardiac troponin level higher than the 99th percentile.

Unstable angina was considered when the patient had a compatible clinical presentation and ECG abnormalities without elevated cardiac troponin level and at least one of the following abnormalities: 1) dynamic changes of the ST-segment within 30 days or during the stress test; 2) a positive test result from stress echocardiography, cardiac MRI, coronary CT angiography; 3) coronary angiography with > 70% occlusion, 4) death within 30 days and, 5) rehospitalisation within 30 days with a diagnosis of ACS.

All patient files were retrospectively analysed by two experts to determine the final diagnosis of ACS or not. Three pairs of experts were recruited from the three centres. They did not belong to the team that included or cared for the patient. Files were randomly assigned. In case of discordance between the two experts, a third one was consulted. The diagnosis was based on pre-hospital data, reports of emergency departments, hospitalization and/or additional examinations, and follow-up on D30.

### Analysis

#### Sample size calculation

At least 10 events per independent variable are recommended to ensure satisfactory statistical power in multivariate regression models [24, 25]. Because we planned to include a maximum of 15 independent variables in the final predictive model for each sex, we needed 150 calls with ACS for men and 150 for women. Given that approximately 16% of calls for non-traumatic chest pain have a definite diagnosis of ACS in the French EMCC, we needed 938 calls for each sex [6]. Given that the percentage of lost to follow-up is estimated to reach 20% (undetermined diagnosis), we needed to include 1123 calls for non-traumatic chest pain for each sex. This sample size estimation concerns the training (derivation) dataset, which corresponds to two-thirds of the overall included patients. Thus, we needed to include a total of 1705 men and 1705 women for the derivation and validation datasets. Owing to an expected sex ratio of women/men of 40%/60%, we needed to include 4263 consecutive calls for non-traumatic chest pain.

#### Analysis

Data are expressed as numbers with percentages for categorial variables or means with standard deviation or medians with interquartile range [IQR] for continuous variables. Categorial data were compared by chi-square or Fisher exact test when appropriate and continuous data by Student *t* or Mann and Whitney test as appropriate. Inter-expert agreement for the final diagnosis was calculated with the Kappa coefficient and its 95% confidence interval.

#### Model development

For each sex, we randomly selected a training (derivation) dataset from two-thirds of the data. Potential predictors were identified as variables associated with an ACS diagnosis significant at *p* < 0.2 on bivariate analysis or already known to be associated in the literature. We used a backward stepwise logistic regression to retain the final independent predictive variables, based on both *p* < 0.05 and the log-likelihood test. Then, we built a receiver operating characteristic (ROC) curve for each sex and defined the area under the ROC curve (AUC).

#### Model validation

The main characteristics of the derivation and validation models were compared for each sex by using appropriate bivariate statistical tests. The internal validity of the predictive model for each sex was tested in the validation dataset. First the discriminative performance of the score was evaluated with the validation dataset. Then, mean predictive probabilities were plotted against observed proportions of ACS in each quintile of predictive probabilities. Differences between observed and predicted probabilities were tested with the Hosmer-Lemeshow test.

All tests were two-sided, with statistical significance set at *p* < 0.05. All analyses were performed with STATA v11.2 (StataCorpLP, College Station, TX, USA) and CART software (Salford System, CA 92126 USA).

## Results

### Characteristics of study subjects

**Fig. 1:**
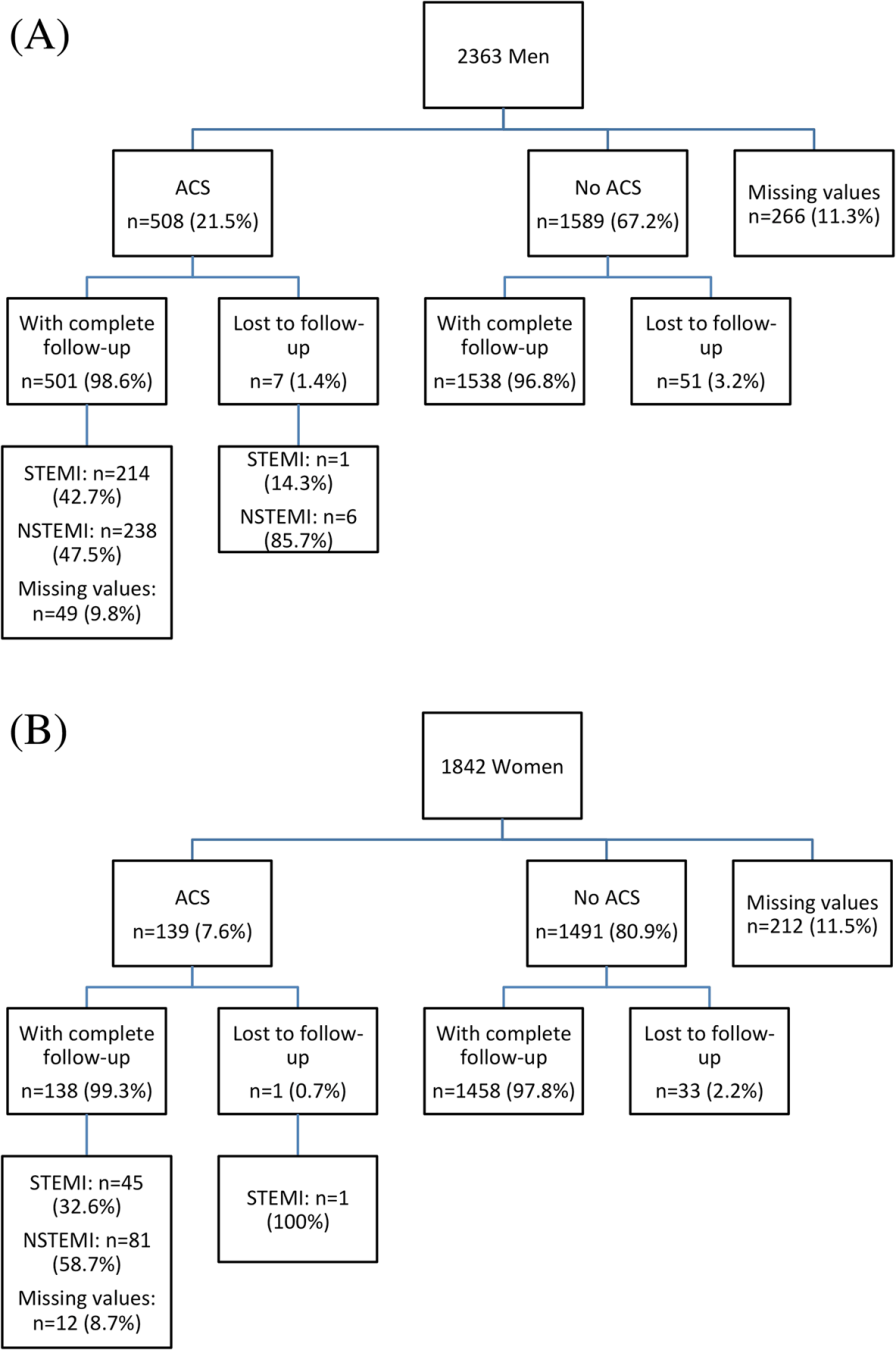
Flowchart based on final diagnosis for men (**a**) and women (**b**). ACS: Acute Coronary Syndrome; NSTEMI: Non ST Elevation Myocardial Infarction; STEMI: ST Elevation Myocardial Infarction

Over the 18 months of the study, 4205 patients were enrolled. A final diagnosis was established for 3727 (89%) (1630 [44%] women and 2097 [56%] men). Flowcharts of the final diagnosis by sex are presented in Fig. 1. Sensitivity analyses are presented in Appendix. Overall, 647 (17%) participants had an ACS diagnosis (508 [24%] men and 139 [9%] women), including 260 (7%) with STEMI. The inter-expert agreement was excellent (Kappa = 0.91 [0.89–0.93]) for diagnosing ACS.

### Main results

#### Males with ACS

The derivation dataset consisted of 1398 men. In total, 324 (23%) men had an ACS diagnosis, 135/324 (42%) with STEMI. The flowchart of the derivation dataset is in the Additional file 2. The general characteristics and type of pain for males are in Table 1. Men with an ACS diagnosis were older, more frequently had cardiovascular risk factors (except family history of coronary diseases and tobacco use) and had more typical pain typography characteristics than those without a diagnosis (Table 1).

**Table 1:** Characteristics and type of pain for male patients in the derivation set with and without a diagnosis of acute coronary syndrome (ACS)

	Total (*n* = 1398)	ACS (*n* = 329)	No ACS (*n* = 1069)	*p* value
Age, mean (SD), y, No. (%)	59.2 (16.1)	59.4 (13.2)	50.9 (16.4)	< 0.001
< 40	291 (20.8)	18 (5.5)	273 (25.5)	< 0.001
40–50	317 (22.7)	59 (17.9)	258 (24.1)	
50–60	320 (22.9)	95 (28.9)	225 (21.0)	
≥ 60	470 (33.6)	157 (47.7)	313 (29.3)	
Coexisting conditions, No. (%)				
Hypertension	405 (29.0)	123 (37.4)	282 (26.4)	< 0.001
Personal coronary artery disease	387 (27.7)	126 (38.3)	261 (24.4)	< 0.001
Family coronary artery disease	231 (16.5)	51 (15.5)	180 (16.8)	0.568
Tobacco use	545 (39.0)	133 (40.4)	412 (38.5)	0.540
Diabetes	183 (13.1)	59 (17.9)	124 (11.6)	0.003
Dyslipidaemia	337 (24.1)	97 (29.5)	240 (22.5)	0.009
Medication therapy, No. (%)				
Aspirin	296 (21.2)	100 (30.4)	196 (18.3)	< 0.001
Clopidogrel	209 (14.9)	77 (23.4)	132 (12.3)	< 0.001
Thyroid hormone	12 (0.9)	2 (0.6)	10 (0.9)	0.743
Statin	263 (18.8)	87 (26.4)	176 (16.5)	< 0.001
Severity of pain, median (IQR)	5 [3–7]	5 [4–7]	5 [3–7]	< 0.001
Type of pain, No. (%)				
Severe pain (NRS ≥6)	592 (42.4)	162 (49.2)	430 (40.3)	0.004
Permanent	350 (25.0)	102 (31.0)	248 (23.2)	0.004
Pain onset:				0.066
Crescendo	307 (22.0)	67 (20.4)	240 (22.5)	
Abruptly	590 (42.2)	157 (47.7)	433 (40.5)	
Unclassified	501 (35.8)	105 (31.9)	396 (37.0)	
Circumstance of pain, No. (%)				0.012
At rest	1106 (79.1)	244 (74.2)	862 (80.6)	
Sport or stress-related	180 (12.9)	58 (17.6)	122 (11.4)	
Unclassified	112 (8.0)	27 (8.2)	85 (8.0)	
Pain typography, No. (%)				
Typical chest pain^a^	799 (57.2)	224 (68.1)	575 (53.8)	< 0.001
Retrosternal	671 (48.0)	201 (61.1)	470 (44.0)	< 0.001
Post-myocardial infarction angina	119/387 (30.8)	50/126 (39.7)	69/261 (24.4)	0.008
Peak type	260 (18.6)	28 (8.5)	232 (21.7)	< 0.001
Burning	182 (13.0)	51 (15.5)	131 (12.3)	0.126
Pinching	81 (5.8)	7 (2.1)	74 (6.9)	0.001
Increasing at position change	252 (18.0)	29 (8.8)	223 (20.9)	< 0.001
Breathing non-related	1037 (74.2)	299 (90.9)	738 (69.0)	< 0.001
Radiating	592 (42.3)	179 (54.4)	413 (38.6)	< 0.001
Additional symptoms	937 (67.1)	249 (75.7)	688 (64.4)	< 0.001

*ACS* Acute coronary syndrome, *NRS* Numeric rating scale, *IQR* Interquartile range

^a^Typical chest pain is characterized by a retrosternal sensation of pressure or heaviness (“angina”), which may be intermittent (usually lasting several minutes) or persistent

Eight factors mostly contributed to the final model for predicting ACS in males: age, tobacco use, severe and permanent pain; retrosternal, breathing non-related and radiating pain; and additional symptoms (Table 2). The AUC value for the final male model was 0.76 (95% CI 0.73–0.79), with no differences between observed and predicted probabilities (Hosmer-Lemeshow test: *p* = 0.78).

**Table 2:** Final model for predicting ACS in males after multivariate analysis

Variables	Regression coefficient	OR	95% CI	*P*-value
Age, y				
< 40	0	1		
40–50	1.085	2.958	[1.668–5.246]	< 0.001
50–60	1.692	5.431	[3.125–9.439]	< 0.001
≥ 60	1.969	7.166	[4.162–12.336]	< 0.001
Tobacco use	0.359	1.432	[1.064–1.927]	0.018
Severe pain (NRS ≥ 6)	−1.016	0.362	[0.143–0.917]	0.032
Permanent pain	0.385	1.469	[1.09–1.981]	0.012
Breathing non-related pain	0.813	2.254	[1.281–3.967]	0.005
Retrosternal pain	0.457	1.580	[1.203–2.075]	0.001
Radiating pain	0.465	1.592	[1.209–2.097]	0.001
Additional symptoms	0.066	1.068	[0.735–1.551]	0.729
Severe pain* Breathing non-related	0.838	2.313	[1.015–5.271]	0.046
Severe pain*Additional symptoms	0.850	2.339	[1.202–4.553]	0.012

Because of missing values for one man, the analyses were performed on 1397 males

*OR* Odds ratio, *CI* Confidence interval, *NRS* Numeric rating scale

The symbol "*" indicate the interaction

**Fig. 2:**
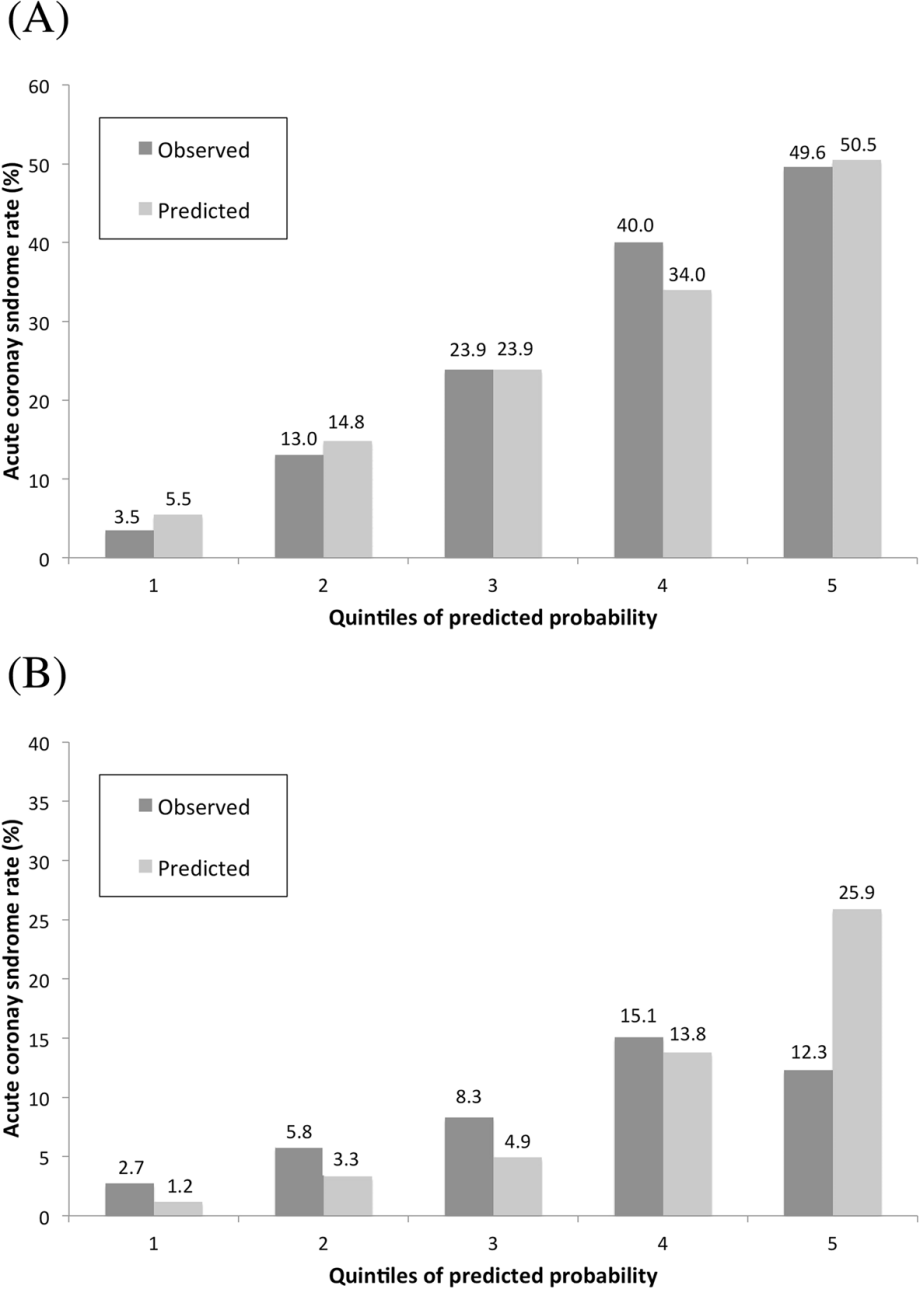
Proportions of acute coronary syndrome cases observed and predicted for males (**a**) and females (**b**)

General characteristics were well balanced between the derivation dataset (*n* = 1398) and the validation dataset (*n* = 699). The accuracy of the male model to predict ACS was validated, with no differences between observed and predicted probabilities (Hosmer-Lemeshow test: *p* = 0.554, Fig. 2a). The AUC value for the male prediction score was 0.76 (0.73–0.80).

#### Females with ACS

The derivation dataset consisted of 1087 women. Overall, 92 (8%) women had an ACS diagnosis, 32/92 (35%) with STEMI. The flowchart of the derivation dataset is in the Additional file 2. The general characteristics and type of pain for females are in Table 3. Women with an ACS diagnosis were older, with more cardiovascular risk factors (except family history of coronary diseases and tobacco use) and had more typical pain typography than those without a diagnosis (Table 3).

**Table 3:** Characteristics and type of pain for female patients in the derivation set with and without a diagnosis of ACS

	Total (*n* = 1087)	ACS (*n* = 92)	No ACS (*n* = 995)	*p* value
Age, mean (SD), y, No. (%)	55.2 (18.1)	69.7 (13.7)	53.9 (17.9)	< 0.001
< 60	215 (19.8)	9 (9.8)	206 (20.7)	< 0.001
≥ 60	445 (40.9)	75 (81.5)	370 (37.2)	
Coexisting conditions, No. (%)				
Hypertension	382 (35.1)	49 (53.3)	333 (33.5)	< 0.001
Personal coronary artery disease	179 (16.5)	35 (38.0)	144 (14.5)	< 0.001
Family coronary artery disease	178 (16.4)	10 (10.9)	168 (16.9)	0.136
Tobacco use	249 (22.9)	12 (13.0)	237 (23.8)	0.019
Diabetes	130 (12.0)	17 (18.5)	113 (11.4)	0.044
Dyslipidaemia	207 (19.0)	28 (30.4)	179 (18.0)	0.004
Medication therapy, No. (%)				
Aspirin	129 (11.9)	24 (26.1)	105 (10.6)	< 0.001
Clopidogrel	64 (5.9)	16 (17.4)	48 (4.8)	< 0.001
Thyroid hormone	90 (8.3)	7 (7.6)	83 (8.3)	0.807
Statin	132 (12.1)	20 (21.7)	112 (11.3)	0.003
Severity of pain, median (IQR)	5 [4–7]	5 [4–7]	5 [4–7]	0.496
Type of pain, No. (%)				
Severe pain (NRS ≥6)	489 (45.0)	42 (45.7)	447 (45.0)	0.900
Permanent	272 (25.0)	24 (26.1)	248 (24.9)	0.805
Pain onset:				0.620
Crescendo	207 (19.1)	14 (15.2)	193 (19.4)	
Abruptly	483 (44.4)	43 (46.7)	440 (44.2)	
Unclassified	397 (36.5)	35 (38.1)	362 (36.4)	
Circumstance of pain, No. (%)				0.210
At rest	914 (84.1)	75 (81.5)	839 (84.3)	
Sport or stress-related	81 (7.5)	5 (5.4)	76 (7.6)	
Unclassified	92 (8.5)	12 (13.1)	80 (8.0)	
Pain typography, No. (%)				
Typical chest pain^a^	616 (56.7)	64 (69.6)	552 (55.5)	0.009
Retrosternal	461 (42.4)	52 (56.5)	409 (41.1)	0.004
Post-myocardial infarction angina	41/179 (22.9)	9/35 (25.7)	32/144 (22.2)	0.659
Peak type	216 (19.9)	6 (6.5)	210 (21.1)	0.001
Burning	132 (12.1)	14 (15.2)	118 (11.9)	0.345
Pinching	53 (4.9)	1 (1.1)	52 (5.2)	0.122
Increasing at position change	229 (21.1)	5 (5.4)	224 (22.5)	< 0.001
Breathing non-related	769 (70.7)	83 (90.2)	686 (68.9)	< 0.001
Radiating	475 (43.7)	52 (56.5)	423 (42.5)	0.010
Additional symptoms	737 (67.9)	70 (76.1)	667 (67.1)	0.077

*ACS* Acute coronary syndrome, *NRS* Numeric rating scale, *IQR* Interquartile range

^a^Typical chest pain is characterized by a retrosternal sensation of pressure or heaviness (“angina”), which may be intermittent (usually lasting several minutes) or persistent

Four factors mostly contributed to the final model for predicting ACS in females: age ≥ 60 years, personal history of coronary artery disease, and breathing non-related and radiating pain (Table 4). The AUC value for the final female model was 0.79 (95% CI: 0.75–0.83), with no differences between observed and predicted probabilities (Hosmer-Lemeshow test: *p* = 0.70).

**Table 4:** Final model for predicting ACS in males after multivariate analysis

Variables	Regression coefficient	OR	95% CI	*P* value
Age ≥ 60 y	1.716	5.564	[3.160–9.800]	< 0.001
Personal history of coronary artery disease	0.603	1.828	[1.120–2.982]	0.016
Breathing non-related pain	1.017	2.765	[1.346–5.678]	0.006
Radiating pain	0.469	1.598	[1.017–2.513]	0.042

Because of missing values for one woman, the analyses were performed on 1086 females

*OR* Odds ratio, *CI* Confidence interval

General characteristics were well balanced between the derivation dataset (*n* = 1087) and the validation dataset (*n* = 543). The female model’s accuracy to predict ACS was not validated: predicted probabilities significantly differed from observed values (Hosmer-Lemeshow test: *p* = 0.035, Fig. 2b). The AUC value for the female prediction score was 0.67 (0.60–0.74).

## Discussion

### Main results

For adults calling an EMCC with chest pain or discomfort, predictors of a final ACS diagnosis differed by sex. The discriminative performance of the model was poor for women and good for men.

### Explanation of the findings

In our study, a predictive variable for ACS in males agreed with traditional typical angina. The pain characteristics were so typical that even the coexisting conditions, such previous coronary artery disease, did not significantly add to the prediction in the multivariate model. Therefore, decision-making in men is based on the characteristics of pain. For females, except for age and personal history of ACS, factors were not related to typical angina. Thus, decision-making in women is mainly based on criteria other than the pain characteristics. The initial presentation of ACS is well known to differ by sex. In contrast to men, women complain of discomfort or pain due to pinching or burning [16–20]. These discrepancies could be due to disparities in pathophysiology and aetiologies [26, 27]. Women with an ACS diagnosis were more likely to have a normal or mild angiographic coronary heart disease [19, 21]. One-tenth of ACS cases involve spontaneous coronary artery dissection, mainly in young women [28]. Myocardial infarction with a non-obstructive coronary artery (MINOCA) occurs mostly in women and includes coronary endothelial dysfunction, myocarditis or Takostubo syndrome [1]. In these pathologies, traditional cardiovascular risk factors have a low implication.

The identification of a woman presenting an ACS remains a challenge. The mortality rate is higher in women than men because of their atypical initial presentation, older age, and less recourse to coronary angiography [21, 29, 30]. When adjusted on the same level of care, mortality is similar between the sexes, which led the authors of one study to advocate for a diagnosis of ACS in women [31, 32]. In our study, the discriminative performance of our model to predict ACS in women was not reproducible. Determinants were limited in number, not typical and above all not reproducible. An explanation is a potential lack of knowledge regarding variables to investigate to detect ACS in women. Currently, women are assessed with the variables used for men. Specific factors in women need to be investigated.

### Strengths

Our study is the first prospective multicentric study to focus on predicting an ACS diagnosis in patients calling an EMCC for chest pain or discomfort. Other studies analysed patients with an established ACS diagnosis and cared for in an emergency department or cardiology department. Furthermore, this large study improves on the small number of studies evaluating the effectiveness of EMCC [33].

### Limitations

We excluded uncommunicative patients because they could experience impending death, an at-risk sign. This state has already been highlighted in the evaluation of imminent delivery at an EMCC [34]. Thus, we investigated ACS as the only outcome without considering other life threatening causes of chest pain. The outcome was established retrospectively by experts, witch could be consider as subjective and potentially biased. Nevertheless, decision was based on medical records that were collected prospectively, limiting this bias. Lastly, in women we failed to propose a model that accurately predicted an ACS diagnosis in the validation sample.

## Conclusions

A sex disparity exists in screening for ACS in people calling an EMCC because of chest pain. A score could be proposed for men. For women, a better understanding of pathophysiology and symptomatology are needed to increase the detection of ACS.

### Supplementary information

**Additional file 1.** Standardized form for gathering data on patient characteristics, cardiovascular risk factors, medical history and clinical presentation.

**Additional file 2.** Flowchart in the derivation set by sex.

## Data Availability

The statistical code and technical processes are available from the time of publication. Appropriate institutional agreements will be required for anonymised participant data transfer. Requests should be made via email to the corresponding author along with an analysis proposal.

## Appendix

**Table 5:** Characteristics and type of pain by availability of diagnosis

	Diagnosis unknown (*n* = 478)	Diagnosis established (*n* = 3727)	*P* value
Female, No. (%)	212 (44.4)	1630 (43.7)	0.789
Age, mean (SD), y, No. (%)	48.1 (16.5)	55.9 (17.0)	**<0.001**
<40	147 (30.8)	764 (20.5)	**<0.001**
40-50	133 (27.8)	804 (21.6)	
50-60	95 (19.9)	790 (21.2)	
≥60	103 (21.5)	1369 (36.7)	
Coexisting conditions, No. (%)			
Hypertension	84 (17.6)	1181 (31.7)	**<0.001**
Personal coronary artery disease	71 (14.9)	857 (23.0)	**<0.001**
Family coronary artery disease	82 (17.2)	603 (16.2)	0.587
Tobacco use	220 (46.0)	1244 (33.4)	**<0.001**
Diabetes	20 (4.2)	462 (12.4)	**<0.001**
Dyslipidaemia	77 (16.1)	838 (22.5)	**0.001**
Medication therapy, No. (%)			
Aspirin	55 (11.5)	624 (16.7)	**0.003**
Clopidogrel	31 (6.5)	410 (11.0)	**0.002**
Thyroid hormone	20 (4.2)	152 (4.1)	0.912
Statin	40 (8.4)	589 (15.8)	**<0.001**
Severity of pain, median (IQR**)**	5 [3–6]	5 [3–7]	**0.032**
Type of pain, No. (%)			
Severe pain (NRS ≥6)	177 (37.1)	1616 (43.4)	**0.009**
Permanent	112 (23.4)	947 (25.4)	0.348
Pain onset:			0.057
Crescendo	113 (23.6)	782 (21.0)	
Abruptly	179 (37.4)	1608 (43.1)	
Unclassified	186 (38.9)	1337 (35.9)	
Circumstance of call, No. (%)			0.733
At rest	382 (79.9)	3026 (81.2)	
Sport or stress related	52 (10.9)	395 (10.6)	
Unclassified	44 (9.2)	306 (8.2)	
Pain typography, No. (%)			
Typical chest pain^a^	231 (48.3)	2121 (56.9)	**<0.001**
Retrosternal	170 (35.6)	1721 (46.2)	
Post-myocardial infarction angina	14/71 (19.7)	241/857 (28.1)	0.127
Peak type	143 (29.9)	701 (18.8)	**<0.001**
Burning	59 (12.3)	478 (12.8)	0.766
Pinching	19 (4.0)	204 (5.5)	0.169
Increasing at position change	119 (24.9)	726 (19.5)	**0.005**
Breathing non-related	311 (65.1)	2738 73.5)	**<0.001**
Radiating	198 (41.4)	1637 (43.9)	0.299
Additional symptoms	302 (63.3)	2521 (67.7)	0.056

*NRS* Numeric rating scale

^a^Typical chest pain is characterized by a retrosternal sensation of pressure or heaviness (‘angina’), which may be intermittent (usually lasting several minutes) or persistent

## Abbreviations

ACS: Acute Coronary Syndrome
AUC: Area Under the ROC Curve
ECG: Electrocardiography
EMCC: Emergency Medical Communication Centre
IQR: Interquartile Range
MICU: Mobile Intensive Care Unit
MINOCA: Myocardial infarction with a non-obstructive coronary artery
ROC: Receiver Operating Characteristic
STEMI: ST Elevation Myocardial Infarction
