# Supplementary material for: Predicting acute coronary syndrome in males and females with chest pain who call an emergency medical communication centre
Source: Scand J Trauma Resusc Emerg Med. 2019 Oct 17;27:92. doi: 10.1186/s13049-019-0670-y (PMC6798370; doi:10.1186/s13049-019-0670-y)
Supplement: Supplementary file 1 — Additional file 1. Standardized form for gathering data on patient characteristics, cardiovascular risk factors, medical history and clinical presentation. [file 13049_2019_670_MOESM1_ESM.docx]

Additional file 1. Standardized form for gathering data on patient characteristics, cardiovascular risk factors, medical history and clinical presentation

**« Medical regulation of emergency calls for chest pain: construction of a probability score for acute coronary syndrome. »**

**PATIENT INCLUSION**

Patients with **Non-traumatic chest pain** (anterior or posterior, inferior or superior, which may be tight, oppressive, embarrassing, tip or burning); **major** (age ≥ 18 years) and calling the **EMCC first** (patient call) or **second** (third party call before the patient) will be included in the study.

Inclusion the **__ __/__ __/** 2 0 **__ __** at **__ __** h **__ __** min

**PATIENT IDENTIFICATION**

EMCC case number : **__ __.__ __**

Initials : Age : **__ __** years Gender ❑ Male

❑ Female

**PAIN DESCRIPTION**

Beginning of the pain motivating the call the**__ __/__ __/__ __ at __ __**h**__ __** min

Inaugural painful episode ❑ Yes Painful episode within 48 hours of the call ❑ Yes

❑ No ❑ No

Permanent pain ❑ Yes Pain onset ❑ Abruptly

❑ No ❑ Crescendo

**COEXISTING CONDITIONS**

Personal coronary artery disease ❑ Yes

❑ No

If yes, Myocardial infarction ❑Yes Angina ❑Yes

❑ No ❑ No

If yes, post myocardial infarction angina ❑ Yes

❑ No

**CARDIOVASCULAR RISK FACTOR**

Tobacco Use ❑ Yes

❑ No ❑ *If no, smoking cessation > 30days*

Diabetes ❑ Yes Dyslipidaemia❑ Yes Hypertension ❑ Yes

❑ No ❑ No ❑ No

Familial coronary artery disease ❑ Yes

❑ No

**MEDICATION THERAPY**

None ❑ Yes

❑ No

Aspirin ❑ Yes Clopidogrel ❑ Yes Statin ❑ Yes Thyroid hormone ❑ Yes

❑ No ❑ No ❑ No ❑ No

❑ *Cessation < 8j* ❑ *Cessation < 8j* ❑ *Cessation < 8j*

**CHEST PAIN TYPOGRAPHY**

***Circumstances***

At rest ❑ Yes Sport or stress related ❑ Yes

❑ No ❑ No

***Topography***

Retrosternal ❑ Yes

❑ No *If no, left* hemithorax ❑ under the left breast ❑

right hemithorax ❑ under the right breast ❑

***Characteristics***

Trans pectoral ❑ Yes Constrictive ❑ Yes Oppression ❑ Yes

❑ No ❑ No ❑ No

Peak type ❑ Yes Burning ❑ Yes Pinching ❑ Yes

❑ No ❑ No ❑ No

Increasing at position change ❑ Yes Breathing related ❑ Yes

❑ No ❑ No

Relieved by the intake of nitrates ❑ Yes

❑ No

Radiating ❑ No

❑ Yes *If Yes,* ❑ left arm ❑ right arm

❑ jaw ❑ dorsal

❑ other :

| Estimated intensity (Numeric rating scale) : | **1** | **2** | **3** | **4** | **5** | **6** | **7** | **8** | **9** | **10** |
| --- | --- | --- | --- | --- | --- | --- | --- | --- | --- | --- |

**ADDITIONAL SYMPTOMS**

Sweeting ❑ Yes Nausea ❑ Yes Vomiting ❑ Yes Syncope ❑ Yes

❑ No ❑ No ❑ No ❑ No

Anxiety ❑ Yes Dizziness ❑ Yes “Imminent death” sensation ❑ Yes

❑ No ❑ No ❑ No
