# Supplementary material for: Predicting acute coronary syndrome in males and females with chest pain who call an emergency medical communication centre
Source: Scand J Trauma Resusc Emerg Med. 2019 Oct 17;27:92. doi: 10.1186/s13049-019-0670-y (PMC6798370; doi:10.1186/s13049-019-0670-y)
Supplement: Supplementary file 2 — Additional file 2. Flowchart in the derivation set by sex. [file 13049_2019_670_MOESM2_ESM.docx]

Additional file 3: Flowchart in the derivation set by sex


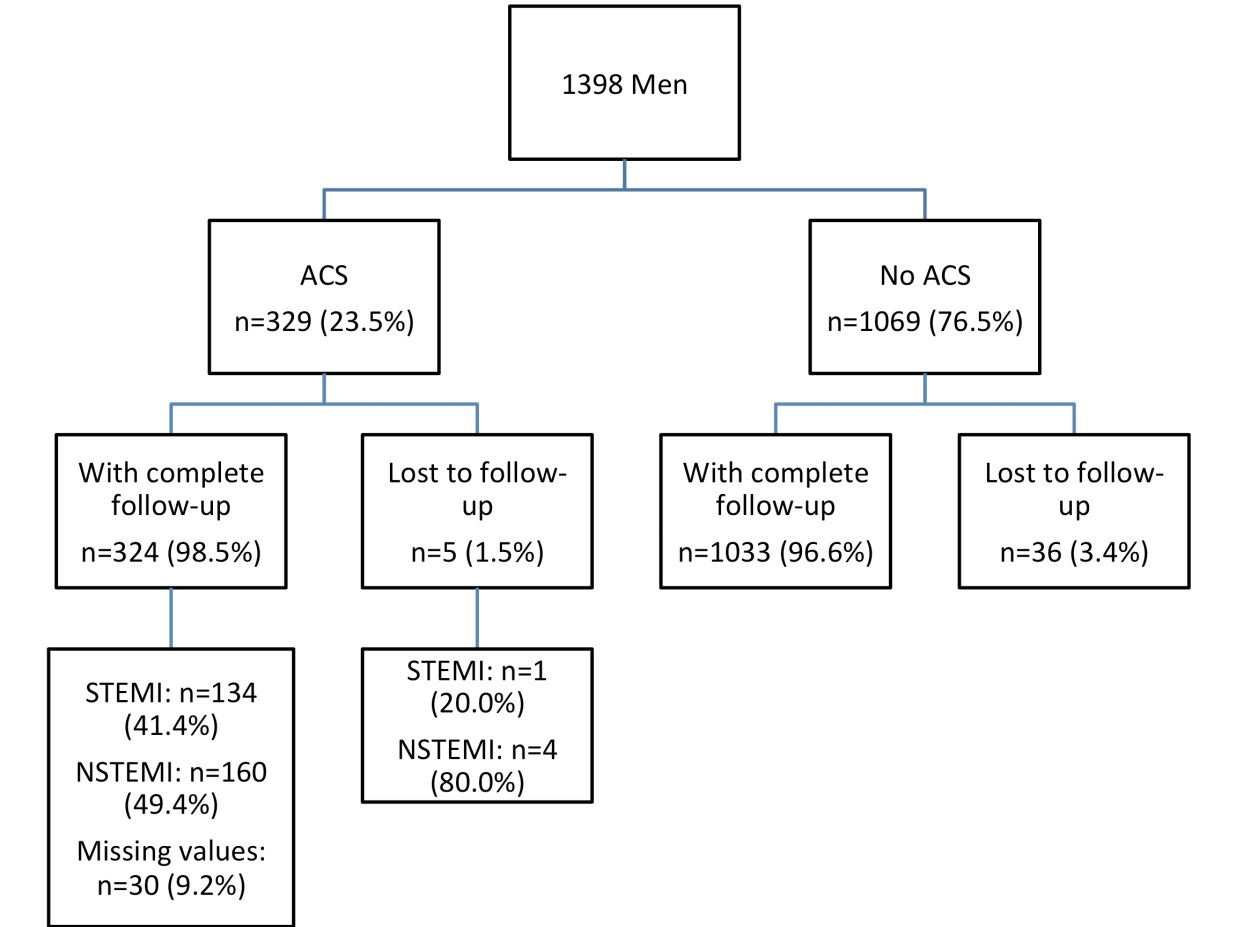


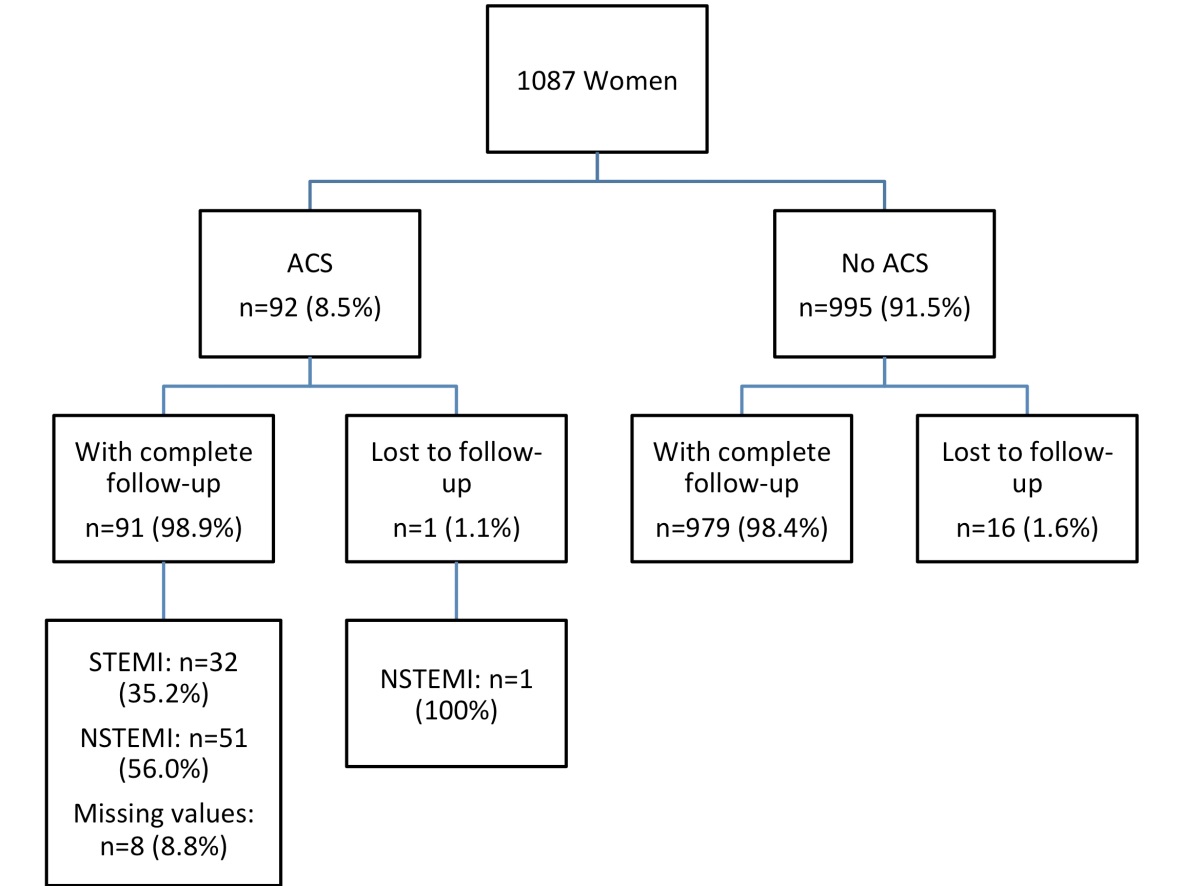


ACS, acute coronary syndrome; STEMI, ST elevation myocardial infarction; NSTEMI, non– ST elevation myocardial infarction
